# Mammographic breast density refines Tyrer-Cuzick estimates of breast cancer risk in high-risk women: findings from the placebo arm of the International Breast Cancer Intervention Study I

**DOI:** 10.1186/s13058-014-0451-5

**Published:** 2014-10-08

**Authors:** Jane Warwick, Hanna Birke, Jennifer Stone, Ruth ML Warren, Elizabeth Pinney, Adam R Brentnall, Stephen W Duffy, Anthony Howell, Jack Cuzick

**Affiliations:** 1 Imperial Clinical Trials Unit, School of Public Health, Faculty of Medicine, Imperial College London, St Mary's Campus, Paddington, London W2 1PG UK; 2 Centre for Cancer Prevention, Wolfson Institute of Preventive Medicine, Queen Mary University of London, Charterhouse Square, London, EC1M 6BQ UK; 3 Centre for Genetic Origins of Health and Disease, University of Western Australia, M40935 Stirling Highway, Perth, WA 6009 Australia; 4 Cambridge Breast Unit, Addenbrooke's Hospital, Hills Road, Cambridge, CB2 0QQ UK; 5 Genesis Breast Cancer Prevention Centre, University Hospital of South Manchester, Southmoor Road, Manchester, M23 9QZ UK

## Abstract

**Introduction:**

Mammographic density is well-established as a risk factor for breast cancer, however, adjustment for age and body mass index (BMI) is vital to its clinical interpretation when assessing individual risk. In this paper we develop a model to adjust mammographic density for age and BMI and show how this adjusted mammographic density measure might be used with existing risk prediction models to identify high-risk women more precisely.

**Methods:**

We explored the association between age, BMI, visually assessed percent dense area and breast cancer risk in a nested case-control study of women from the placebo arm of the International Breast Cancer Intervention Study I (72 cases, 486 controls). Linear regression was used to adjust mammographic density for age and BMI. This adjusted measure was evaluated in a multivariable logistic regression model that included the Tyrer-Cuzick (TC) risk score, which is based on classical breast cancer risk factors.

**Results:**

Percent dense area adjusted for age and BMI (the density residual) was a stronger measure of breast cancer risk than unadjusted percent dense area (odds ratio per standard deviation 1.55 versus 1.38; area under the curve (AUC) 0.62 versus 0.59). Furthermore, in this population at increased risk of breast cancer, the density residual added information beyond that obtained from the TC model alone, with the AUC for the model containing both TC risk and density residual being 0.62 compared to 0.51 for the model containing TC risk alone (*P* =0.002).

Approximately 16% of controls and 19% of cases moved into the highest risk group (8% or more absolute risk of developing breast cancer within 10 years) when the density residual was taken into account. The net reclassification index was +15.7%.

**Conclusions:**

In women at high risk of breast cancer, adjusting percent mammographic density for age and BMI provides additional predictive information to the TC risk score, which already incorporates BMI, age, family history and other classic breast cancer risk factors. Furthermore, simple selection criteria can be developed using mammographic density, age and BMI to identify women at increased risk in a clinical setting.

**Clinical trial registration number:**

http://www.controlled-trials.com/ISRCTN91879928 (Registered: 1 June 2006).

**Electronic supplementary material:**

The online version of this article (doi:10.1186/s13058-014-0451-5) contains supplementary material, which is available to authorized users.

## Introduction

Mammographic density is well-established as a risk factor for breast cancer [1]. Nevertheless, it is not yet widely used to assess breast cancer risk because the established methods of density assessment are not viable for use in large numbers of mammograms, for example, within a national screening programme. A number of fully automated methods have been developed however, which, once validated, are likely to allow large numbers of mammograms to be rapidly and reliably measured for density. A key question, therefore, is `how can we use this information to identify and inform women at greatest risk of developing breast cancer, so that we may offer them risk-reducing interventions?'

Aside from the challenges involved in measuring mammographic density, the issue of how to utilise mammographic density information to estimate breast cancer risk is also complicated by the fact that there is confounding between percent mammographic density, body mass index (BMI) [2] and age [3], and possibly other breast cancer risk factors as well. Thus, to assess a woman's risk of developing breast cancer from her mammographic density, one must take into account her age and BMI [4]. This means that we need a more nuanced approach than using a fixed cut-off of, say, 50% dense. Furthermore, it is not only the dense tissue itself that confers the breast cancer risk. Body fat synthesises and releases estrogens, particularly in postmenopausal women, so that increased BMI is associated with increased breast cancer risk in postmenopausal women [2]. Currently, a woman's risk of developing breast cancer can be assessed using one of a number of established statistical models, but there has been little validation in the general population [5]-[7]. Furthermore, attempts to extend existing models to include mammographic density have so far been disappointing. The incorporation of mammographic density measured by Breast Imaging Reporting and Data System (BI-RADS) categories into the Gail model by Tice *et al.*[8] improved the predictive power minimally. The addition of mammographic density to the Breast Cancer Surveillance Consortium (BCSC) [9], Barlow [10] and Gail [11] models led to only modest improvements in discriminatory power. Nevertheless, in many health care systems access to breast cancer risk-reducing interventions is contingent upon having a high breast cancer risk as assessed by one of these models. It is vital, therefore, to further develop these models to incorporate mammographic density so that when we are making such, sometimes life changing, decisions we are taking into account all available information. In this study, we use data on a subset of women from the placebo arm of the International Breast Cancer Intervention Study 1 (IBIS-1) [12],[13] to develop a model to adjust mammographic density (percent dense area) for age and BMI and show how this adjusted mammographic density measure might be used with existing risk prediction models to identify high-risk women more precisely.

## Materials and methods

The subjects of this study are 558 women from the placebo arm of the IBIS-I, a randomized trial of tamoxifen versus placebo in women at high risk of developing breast cancer. The IBIS-I trial is registered with controlled-trials.com as ISRCTN91879928, which has been reported in full elsewhere [12],[13]. The selection process for this particular subset was described in detail in the report of the IBIS-I density case-control study [14]. There was no matching. Briefly, women were eligible to participate in IBIS-I if they were aged 35 to 70 years and breast cancer free but with their risk of developing the disease estimated to be at least twice the population average. At entry to the study, women were randomised to take either tamoxifen (20 mg daily) or placebo for five years, with six-monthly follow-up appointments. Additionally, mammograms were required at entry to the study and recommended at 12- to 18-month intervals during the treatment period.

Written informed consent to participate in the study and allow access to medical records was obtained from all participants in IBIS-I prior to entry. Further written consent for their mammograms to be used was obtained subsequently. Both IBIS-I and the mammography study were approved by North Somerset and South Bristol Research Ethics Committee. For the IBIS-I density case-control study the mammograms relating to 942 controls (women without breast cancer) and 123 cases (British and Finnish participants diagnosed with breast cancer prior to 1 October 2007) were retrieved from local centres and mammographic density (percent dense area) measured centrally by RW (a consultant radiologist). Film mammograms of the left and right mediolateral oblique views were placed together on the light-box and read as a single entity. Percent dense area was assessed visually to the nearest 5%.

Only participants from the placebo arm of the IBIS-I density study (72 cases, 486 controls) were used in this study as tamoxifen itself has a major impact on both mammographic density and breast cancer development. The majority of cases (64/72) were invasive. Median follow-up for controls was 11.6 years. Median time to diagnosis for cases was 5.1 years.

### Statistical methods

Logistic regression (with breast cancer status as the dependent variable and age, baseline percent dense area and BMI as independent variables) was used to evaluate the association between breast cancer risk and age, BMI and percent dense area.

Linear regression, fitted by ordinary least squares with percent dense area at baseline as the dependent variable and age and BMI as independent variables, was used to adjust percent dense area for age and BMI. The transformation *d* = *log(z/(1-z))*, where *z = [0.025 + 0.95{x-min(x)}/max(x)]*^*1/2*^ and *x* = percent dense area, was used to ensure that the residuals followed an (approximately) normal distribution. Age and BMI were centred for interpretability. Adjusted percent dense area (density residual) was calculated as the difference between the observed percent dense area (after transformation) and the fitted value. Density residuals were standardised to have mean 0 and variance 1. To be certain that all relevant breast cancer risk factors had been included in the density adjustment, we also investigated the effect of adding age at menarche, parity, menopausal status, previous biopsy, hormone replacement therapy, and atypical hyperplasia or/and lobular carcinoma *in situ* to the model.

In order to explore the additional effect on estimates of breast cancer risk of adding density residual to a breast cancer risk assessment model based on standard breast cancer risks factors, we fitted a logistic regression model with density residual and absolute risk of developing breast cancer within 10 years (as computed by the Tyrer-Cuzick (TC) model [15]) as independent variables and breast cancer status as the dependent variable. The Tyrer-Cuzick model incorporates familial and personal risk factors (including those listed above) but does not so far include mammographic density. For comparison, we also fitted a univariate logistic regression model with unadjusted percent dense area as the independent variable. Likelihood ratio tests were used to assess whether the addition of each variable improved discrimination [16]. All *P* values were two-sided.

For each subject the absolute risk of developing breast cancer within the next 10 years (<3%, 3 to 5%, 5 to 8%, 8 +%) was calculated first from the TC model then modified to reflect the effect of mammographic density (by multiplying the TC risk by the predicted odds ratio from the logistic regression model containing only the density residual or only the unadjusted density). The net reclassification index for the TC model compared to TC plus density residual, and for the TC model compared to TC plus unadjusted density, was calculated and re-classification tables presented [17].

Agreement between density readings obtained at different time points, or by different readers, was assessed using Lin's concordance correlation coefficient [18],[19].

## Results

The number of cases and controls, the total years follow-up and baseline characteristics of the study group are shown in Table 1. Median follow-up for controls was 11.6 years. Median time to diagnosis for cases was 5.1 years. The TC risk score (absolute risk of developing breast cancer within the next 10 years) for our study group varied from 1.6% to 34.2% with mean 6.0% (median 5.5%). Median percent dense area was 42.5% in controls (interquartile range (IQR) 15.0 to 70.0) and 62.5% (IQR 25.0 to 80.0) in cases. BMI was missing for six control women, age at menarche for four women (two controls and two cases) and previous biopsy for one control woman.

**Table 1:** **Baseline characteristics of the study group (72 breast cancer cases and 486 controls from the placebo arm of IBIS-I)**

Variable	Controls	Cases
	(n =486)	(n =72)
**Follow-up (years)** ^**†**^	11.6 (10.7-12.3)	5.1 (3.1-7.9)
**Age (years)** ^**†**^	49.5 (46.4-54.4)	49.9 (46.3-56.0)
**BMI (kg/m** ^**2**^ **)** ^**†**^	25.8 (23.4-29.1)	26.2 (23.7-28.4)
**Menopausal status**		
Pre/peri	261 (54%)	38 (53%)
Post	225 (46%)	34 (47%)
**HRT use**		
Never	316 (65%)	46 (64%)
Current	108 (22%)	14 (19%)
Previous	62 (13%)	12 (17%)
**Age at menarche (years)** ^**†**^	13 (12-14)	13 (12-14)
**Age at first birth**		
**Nulliparous**	69 (14%)	12 (17%)
>29 years	55 (11%)	6 (8%)
26-29 years	98 (20%)	14 (19%)
21-25 years	178 (37%)	27 (38%)
<21 years	86 (18%)	13 (18%)
**Tyrer-Cuzick risk score**		
10-year absolute risk (%)^†^	5.5 (4.5-6.7)	5.4 (4.1-6.9)
10 -year relative risk^†^	2.2 (1.8-2.7)	2.1 (1.7-2.9)
**Mammographic density**		
Percent dense area (%)^†^	42.5 (15.0-70.0)	62.5 (25.0-80.0)

^†^Median (interquartile range). BMI, body mass index; HRT, hormone replacement therapy.

The reproducibility of the density readings was assessed by having the images re-read by RW and another trained reader (JS) 10 years after the initial reading. For the baseline mammograms the concordance coefficient between the original and repeat readings by the original reader (RW) was very high (0.88 95% confidence interval (CI): 0.87 to 0.90) with an average difference between readings of −3.04. The concordance coefficient between the original readings by RW and repeat readings by JS was also very high (0.88 95% CI: 0.87 to 0.90) with an average difference between readings of 4.42.

The odds ratios for risk of developing breast cancer from the multivariate logistic regression model including age, BMI and percent dense area were 1.33 (95% CI: 0.86 to 2.04, *P* =0.20) per 10-year change in age at entry to IBIS-I, 1.05 (95% CI: 0.99 to 1.11, *P* =0.07) per one unit of BMI (kg/m^2^), and 1.16 (95% CI: 1.06 to 1.27, *P* =0.001) per 10% change in percent dense area.

Details of how the density residual, which ranged from −2.74 to 2.79, was calculated are given in Appendix A.

The odds ratios, confidence intervals, area under the receiver operating characteristic curve (AUC) and associated *P* values from the univariate and multivariate logistic regression models with absolute TC risk, unadjusted percent dense area and density residual as independent variables and breast cancer status as the dependent variable are reported in Table 2. The density residual was a stronger measure of breast cancer risk than unadjusted percent dense area (odds ratio per standard deviation 1.55 vs. 1.38) with the AUC being 0.62 compared with 0.59. The density residual added statistically significant information beyond that obtained from the TC model alone (*P* =0.002). Unadjusted percent dense area was not significant in a model that already included the density residual.

**Table 2:** **Odds ratios (OR) and area under the curve (AUC) from the logistic regression models including absolute Tyrer-Cuzick (TC) risk score, unadjusted percent dense area and density residual as independent variables**

Model	Odds ratio^†^(95% CI)	AUC	LR-CHI2	Δ χ^2^(***P***value)^a^
**Univariate**				
Absolute TC risk score	1.18 (10.99-1.39)	0.51	3.5	0.06
Unadjusted percent dense area	1.83 (1.14-2.94)	0.59	6.5	0.01
Density residual	1.90 (1.31-2.77)	0.62	11.7	0.001
**Multivariate**				
Absolute TC risk	1.12 (0.94-1.33)		3.5	0.06
Density residual	1.82 (1.25-2.67)	0.62	9.9	0.002

^†^Odds ratio is for the difference between the 75^th^ and 25^th^ percentiles of the Tyrer-Cuzick (TC) 10-year absolute risk, unadjusted percent dense area and the density residual; ^a^this is the change in χ^2^ when the variable is added to the model.

The numbers reclassified (absolute risk of developing breast cancer within the next 10 years 3%, 3 to 5%, 5 to 8%, 8 +%) when the TC risk score was modified using the density residual are given in Table 3. The net reclassification index is +15.7%. Approximately 16% of controls (76/480) moved into the highest risk group (8 +%) when the density residual was taken into account and 4% (19/480) moved from the highest risk group to a lower risk one. Amongst cases, the equivalent figures were 19% (14/72) and 1% (1/72) respectively. For comparison, the numbers reclassified when unadjusted percent dense area is used to modify the TC risk score rather than density residual are also presented in Table 4. Approximately 33% of controls (157/480) moved from the highest risk group (8 +%) to a lower risk group when the density residual was used to modify TC risk rather than percent dense area. Amongst cases, the equivalent figure was 21% (15/72) respectively. The net reclassification index is 16.9%.

**Table 3:** **Numbers reclassified (absolute risk of developing breast cancer within the next 10 years 3%, 3 to 5%, 5 to 8%, 8 +%) for the Tyrer-Cuzick (TC) risk score compared to Tyrer-Cuzick risk score modified using the density residual**

TC/TC + density residual	<3%	3-5%	5-8%	8%+	Total
**Control**					
<3%	10 (67%)	4 (27%)	1 (7%)	0 (0%)	15 (3%)
3-5%	52 (30%)	65 (38%)	44 (26%)	10 (6%)	171 (36%)
5-8%	8 (3%)	68(29%)	92 (39%)	66 (28%)	234 (49%)
8 +%	0 (0%)	3 (5%)	16 (27%)	41 (68%)	60 (12%)
**Total**	70 (15%)	140 (29%)	153 (32%)	117 (24%)	480 (100%)
**Case**					
<3%	2 (50%)	2 (50%)	0 (0%)	0 (0%)	4 (6%)
3-5%	5 (17%)	13 (43%)	7 (23%)	5 (17%)	30 (42%)
5-8%	0 (0%)	9 (39%)	5 (22%)	9 (39%)	23 (32%)
8 +%	0 (0%)	0 (0%)	1 (7%)	14 (93%)	15 (21%)
**Total**	7 (10%)	24 (33%)	13 (18%)	28 (39%)	72 (100%)

**Table 4:** **Numbers reclassified (absolute risk of developing breast cancer within the next 10 years 3%, 3 to 5%, 5 to 8%, 8 +%) for the Tyrer-Cuzick (TC) risk score modified using percent dense area compared to the Tyrer-Cuzick risk score modified using the density residual**

TC + unadjusted density/TC + density residual	<3%	3-5%	5-8%	8%+	Total
**Control**					
<3%	4 (100%)	0 (0%)	0 (0%)	0 (0%)	4 (1%)
3-5%	47 (89%)	6 (11%)	0 (0%)	0 (0%)	53 (11%)
5-8%	19 (13%)	105 (70%)	25 (17%)	0 (0%)	149 (31%)
8 +%	0 (0%)	29 (10%)	128 (47%)	117 (43%)	274 (57%)
**Total**	70 (15%)	140 (29%)	153 (32%)	117 (24%)	480 (100%)
**Case**					
<3%	0 (0%)	0 (0%)	0 (0%)	0 (0%)	0 (0%)
3-5%	6 (55%)	5 (45%)	0 (0%)	0 (0%)	11 (15%)
5-8%	1 (6%)	15 (83%)	2 (11%)	0 (0%)	18 (25%)
8 +%	0 (0%)	4 (9%)	11 (26%)	28 (65%)	43 (60%)
**Total**	7 (10%)	24 (33%)	13 (18%)	28 (39%)	72 (100%)

## Discussion

We have shown that by adjusting percent dense area for age and BMI a better measure of breast cancer risk is obtained. This adjusted measure provided additional predictive information when added to the TC risk estimates calculated from classic breast cancer risk factors. Our findings suggest that even within known high-risk groups, prevention strategies might be better targeted as, with the addition of information from the density residual, the number of women identified as having the highest breast cancer risk (>8%) increased from 14% (75/552) to 26% (145/552) and the proportion of cases arising in the highest risk group increased from 21% (15/72) to 39% (28/72). We also found that a woman with high density residual but low TC risk might have a greater chance of developing breast cancer than a similar woman with low density residual but high TC risk. Furthermore, the density residual was better at modifying TC risk than unadjusted percent dense area.

Our study has a number of limitations. First, our model for adjusting percent dense area for age and BMI is based on a relatively small number of women and therefore requires validation. Second, since our subjects are from a higher-risk population, our adjustment may not be appropriate for use in the general population. This may also explain the poor discrimination of the model containing TC risk alone. Nevertheless, our results highlight the fact that even among high-risk women the addition of mammographic density to existing breast cancer risk prediction models seems likely to improve discrimination. The further development and validation of these models should therefore be a priority.

A weakness of previous attempts to incorporate information on breast density into established risk prediction models is that the only available measure of breast density was categorical and therefore a less sensitive measure. In our study group including breast density adjusted for age and BMI improved the predictive ability of the model. The model with TC risk and density residual had almost four times as much information on breast cancer risk as the TC risk alone (χ^2^ = 13.4 vs. 3.50). Therefore, an important step will be to develop the TC model further by incorporating an adjusted density measure into the model. Further validation of these results in the Predicting Risk Of breast Cancer At Screening (PROCAS) study [20] is planned as well as further analyses of the relation between adjusted density and other classic risk factors. Previous publications [8]-[10] on incorporation of breast density into risk assessment models mainly used BI-RADs categories. Chen *et al*. [11] used a continuous measure of percent dense area coded into four categories, while we used percent dense area assessed to the nearest 5%. Therefore, a standardised breast density reporting must be achieved before breast density can be incorporated into a clinically useful risk assessment model [21].

## Conclusions

We have found adjusting percent dense area for age and BMI gives a stronger and more independent measure of breast cancer risk. Adjusted density adds information to a risk score from the TC model that already incorporates BMI, age, family history and other risk factors. Furthermore, simple selection criteria can be developed using mammographic density, age and BMI to identify women at increased risk in a clinical setting.

## Authors' contributions

JW designed the study, analysed the data, interpreted the results and drafted the paper. HB analysed the data, interpreted the results and drafted the paper. JS designed the study, interpreted the results and reviewed the paper. RMLW read the mammograms, interpreted the results and reviewed the paper. EP collected the mammograms and drafted the paper. ARB analysed the data, interpreted the results and reviewed the paper. SWD designed the study, interpreted the results and reviewed the paper. AH interpreted the results and reviewed the paper. JC designed the study, interpreted the results and drafted the paper. All authors read and approved the final manuscript.

## Appendix A

### Calculation of the density residual

Age, BMI, menopausal status, parity, age at menarche, use of hormone replacement therapy, previous biopsy and atypical hyperplasia or/and lobular carcinoma *in situ* were considered for entry to the linear regression model with transformed percent dense area (calculated as *log*(*z*/(1 - *z*)), *where z* = [0.025 + 0.95{*x* - *min*(*x*)}/*max*(*x*)]^1/2^ and *x* = percent dense area) as the dependent variable. The use of hormone replacement therapy and age at menarche was not significant. BMI explained around 15% of the variation in transformed percent dense area, age explained 5%, parity explained 0.8%, menopausal status 0.6%, previous biopsy 1.8% and atypical hyperplasia or/and lobular carcinoma *in situ* 1.3%. Compared with age and BMI, the additional explanatory value of the other risk factors was quite low so we decided not to include them in the adjustment.

Adjusted percent dense area was calculated from the following linear regression line

$$0.9208-0.1156\;x\;\left(BMI-26\;\mathrm{kg}/m^{2}\right)\;–\;0.0542\;x\;\left(\mathrm{Age}-50\;\mathrm{yr}\right)$$

Density residual was calculated as:

*transformed percent dense area-adjusted percent dense area.*

To standardise, the density residual was divided by its standard deviation (1.30).

## Authors’ original submitted files for images

Below are the links to the authors’ original submitted files for images. Authors’ original file for figure 1 Authors’ original file for figure 2

## Abbreviations

AUC: area under the receiver operating characteristic curve
BI-RADS: Breast Imaging Reporting and Data System
BMI: body mass index
CI: confidence interval
HRT: hormone replacement therapy
IBIS-I: International Breast Cancer Intervention Study I
IQR: interquartile range
TC: Tyrer-Cuzick
