# Supplementary material for: Mammographic breast density refines Tyrer-Cuzick estimates of breast cancer risk in high-risk women: findings from the placebo arm of the International Breast Cancer Intervention Study I
Source: Breast Cancer Res. 2014 Oct 8;16:451. doi: 10.1186/s13058-014-0451-5 (PMC4303130; doi:10.1186/s13058-014-0451-5)
Supplement: Supplementary file 1 [file 13058_2014_451_MOESM1_ESM.docx]

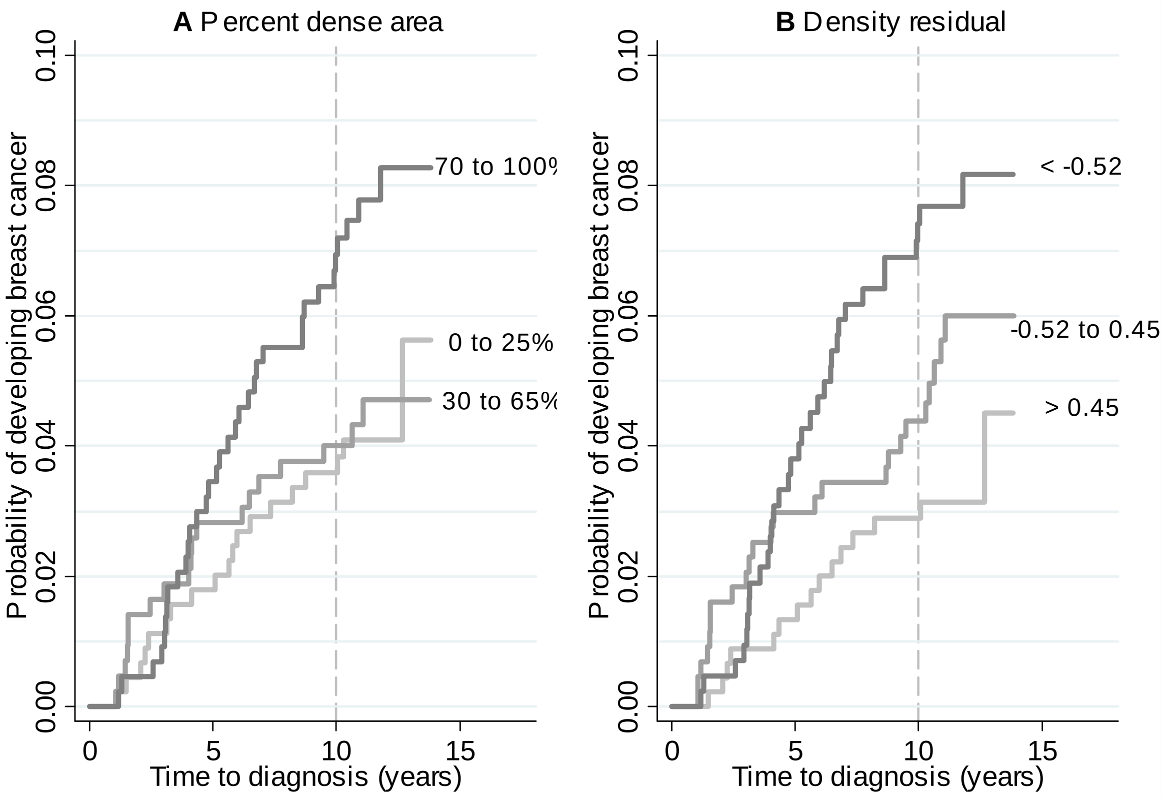
A Percent dense area
B Density residual
Probability of developing breast cancer
0.00
0.02
0.04
0.06
0.08
0.10
0
5
10
15
Time to diagnosis (years)
70 to 100%
0 to 25%
30 to 65%
< -0.52
-0.52 to 0.45
> 0.45
