# Supplementary material for: Mammographic breast density refines Tyrer-Cuzick estimates of breast cancer risk in high-risk women: findings from the placebo arm of the International Breast Cancer Intervention Study I
Source: Breast Cancer Res. 2014 Oct 8;16:451. doi: 10.1186/s13058-014-0451-5 (PMC4303130; doi:10.1186/s13058-014-0451-5)
Supplement: Supplementary file 2 [file 13058_2014_451_MOESM2_ESM.docx]

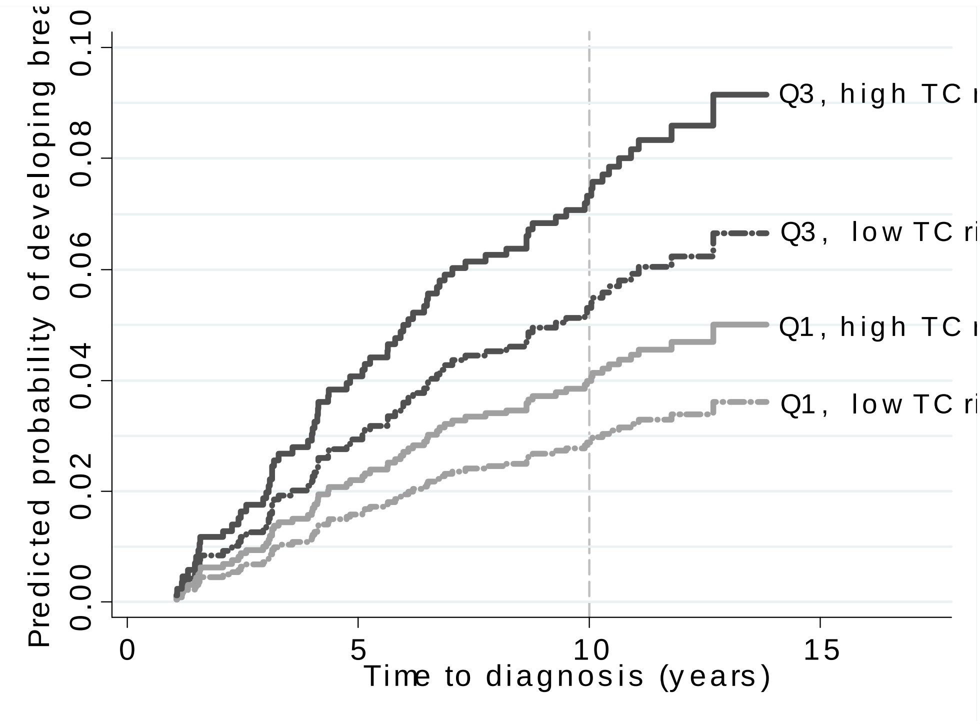

Predicted probability of developing brea
0.00
0.02
0.04
0.06
0.08
0.10
0
5
10
15
Time to diagnosis (years)
Q3, high TC r
Q3, low TC ri
Q1, high TC r
Q1, low TC ri
